# Particular distribution and expression pattern of endoglin (CD105) in the liver of patients with hepatocellular carcinoma

**DOI:** 10.1186/1471-2407-7-122

**Published:** 2007-07-04

**Authors:** Decai Yu, Linyuan Zhuang, Xitai Sun, Jun Chen, Yongzhong Yao, Kui Meng, Yitao Ding

**Affiliations:** 1 Institute of Hepatobiliary Surgery, Nanjing University, Nanjing, Jiangsu Province, PR China; 2 Department of Hepatobiliary Surgery, the Affiliated Drum Tower Hospital, School of Medicine, Nanjing University, Nanjing, Jiangsu Province, PR China; 3 Department of Pathology, the Affiliated Drum Tower Hospital, School of Medicine, Nanjing University, Nanjing, Jiangsu Province, PR China

## Abstract

**Background:**

Endoglin (CD105) has been considered a prognostic marker for hepatocellular carcinoma (HCC), and widely used as an appropriate targeting for antiangenesis therapy in some cancers. Our aim was to evaluate the distribution and expression of CD105 in the liver of patients with HCC, and to discuss whether CD105 may be used as an appropriate targeting for antiangenesis therapy in HCC.

**Methods:**

Three parts of liver tissues from each of 64 patients with HCC were collected: tumor tissues (TT), adjacent non-tumor (AT) liver tissues within 2 cm, and tumor free tissues (TF) 5 cm far from the tumor edge. Liver samples from 8 patients without liver diseases served as healthy controls (HC). The distribution and expression of CD105 in tissues were evaluated by immunohistochemistry, Western blotting analysis, and real-time PCR. HIF-1alpha and VEGF_165 _protein levels in tissues were analyzed by Immunohistochemistry and Western blotting analysis or ELISA.

**Results:**

CD105 was positively stained mostly in a subset of microvessels 'endothelial sprouts' in TT of all patients while CD105 showed diffuse positive staining, predominantly on hepatic sinus endothelial cells in the surrounding of draining veins in TF and AT. The mean score of MVD-CD105 (mean ± SD/0.74 mm^2^) was 19.00 ± 9.08 in HC, 153.12 ± 53.26 in TF, 191.12 ± 59.17 in AT, and 85.43 ± 44.71 in TT, respectively. Using a paired *t *test, the expression of CD105 in AT and TF was higher than in TT at protein (MVD, *p *= 0.012 and *p *= 0.007, respectively) and mRNA levels (*p *< 0.001 and *p *= 0.009, respectively). Moreover, distribution and expression of CD105 protein were consistent with those of HIF-1alpha and VEGF_165 _protein in liver of patients with HCC. The level of *CD105 *mRNA correlated with VEGF_165 _level in TF (r = 0.790, *p *= 0.002), AT (r = 0.723, *p *< 0.001), and TT (r = 0.473, *p *= 0.048), respectively.

**Conclusion:**

It is demonstrated that CD105 was not only present in neovessels in tumor tissues, but also more abundant in hepatic sinus endothelium in non-tumor tissues with cirrhosis. Therefore, CD105 may not be an appropriate targeting for antiangenesis therapy in HCC, especially with cirrhosis.

## Background

Endoglin (CD105) is a homodimeric transmembrane glycoprotein highly expressed on activated endothelial cells, and is involved in vascular development and remodeling [1,2]. In line with these findings, compared to the conventional biomarker CD34, CD105 has been demonstrated to be a superior angiogenesis marker in breast cancer [3], malignant melanoma [4], non-small cell lung cancer [5], and colorectal carcinoma [6]. These findings have provided supportive evidence to the usefulness of CD105 targeting in antiangiogenetic therapy of cancer [7,8]. Seon's studies have demonstrated long-lasting complete abrogation of human breast tumors in SCID mice using CD105 antibody with immunotoxins [9,10] and growth suppression of human solid tumors using radiolabeled antibody to CD105 [11]. In a clinical investigation, Costello et al reported that^ 99^Tc^m^-labeled antibody to CD105 had the ability of the specific localization in the tumor vasculature of the kidneys [12].

As a typical hypervascular tumor, hepatocellular carcinoma (HCC) is the most common hepatic malignancy worldwide, especially in South-east Asia. Approximately 80% of HCC patients have been associated with liver cirrhosis [13]. Even after comprehensive therapies with surgical excision, chemotherapy, ethanol injection, radiofrequency, or cryotherapy, this tumor shows a high percentage of recurrence and metastasis, and the mean survival of the patients is still short, compared to other major solid tumors. It is assumed that such high vascularity could be one of the reasons responsible for the poor prognosis [14]. Innovative approaches, such as targeting the non-transformed, less resistant, tumor supporting endothelial cells, may change this outcome [15]. Our previous investigation demonstrated the superiority of CD105 to CD34 as a marker of angiogenesis in HCC, which was consistent with the investigation of Ho [16,17]. Therefore, we hypothesized that CD105 might be an appropriate targeting for antiangiogenesis therapy in HCC.

To validate the specificity of targeting for antiangenesis therapy with CD105 in HCC, we further evaluated the distribution and expression of CD105 in liver with HCC at protein and mRNA levels. Moreover, two relevant factors with the expression of CD105, hypoxia inducing factor 1alpha (HIF-1alpha) and the 165-amino acid form of vascular endothelial growth factor (VEGF_165_), were also evaluated at protein level.

## Methods

### Patients and tissue specimens

Sixty-four HCC patients, hospitalized in the Department of Hepatobiliary Surgery of Drum Tower Hospital between January 2004 and August 2006, were enrolled in the present study. None of the patients had received preoperative treatment, such as transarterial chemoembolization, et al. Normal liver tissues from 4 donors for liver transplantation and 4 patients with no evidence of liver diseases severed as healthy controls (HC). The research ethics committee of Drum Tower hospital approved this protocol and verbal consent was obtained from all participants.

Tumor tissues (TT), adjacent non-tumor tissues (AT) within 2 cm, and tumor free tissues (TF) more than 5 cm far from the tumor edge were collected immediately after surgical resection from each of the 64 HCC patients as described by Mathonnet [18]. Necrotic or hemorrhagic tissues were excluded. Tissues were snap-frozen and kept in liquid nitrogen until use (protein and RNA isolation), or were fixed in 10% formalin and embedded in paraffin for immunohistochemical study and Hematoxylin and Eosin stain (H&E). Four μm thick sections were prepared and stained with H&E for study of the pathological features of HCC in accordance with the Classification of Carcinomas of the Liver proposed by UICC [19]. Histopathological examination was evaluated by a senior pathologist (Prof. Zhang), who was unaware of the results of this study. Serial sections of the tumors and surrounding tissues were examined to identify any tumor encapsulation, microscopic venous invasion, and microsatellite lesions.

### Immunohistochemical staining for CD105, HIF-1alpha and VEGF_165_

Consecutive paraffin sections from HC, TF, AT, and TT were immunoassayed with the antibodies to human CD105 (1:400, H-300, Santa Cruz, CA), HIF-1alpha (1:400, Chemicon, CA), and VEGF_165 _(1:100, Santa Cruz). A subsequent reaction was performed with biotin-free HRP enzyme labeled polymer from an En Vision plus detection system (DAKO, CA). Positive reactions were visualized with diaminobenzidine (DAB) solution followed by counterstaining with hematoxylin. Negative controls were performed using non-immune goat serum instead of the primary antibodies.

Microvessel density (MVD) was evaluated according to Gasparini's criteria by two independent investigators as described in previous reports [20,21]. The mean microvessel count of the five most vascular areas was taken as the MVD, which was expressed as the absolute number of microvessels per 0.74 mm^2 ^(×200 field) on an Olympus microscope (CX-31) with an Olympus camera (C-5050Z). For HIF-1alpha and VEGF_165 _analysis, 10 areas were randomly selected and counted under a microscope at a magnification of 200. According to the degree of distribution of immunoreactive cells, HIF-1alpha and VEGF_165 _expression were graded into three levels: negative when the stained cells were present in less than 10% of the entire area; weakly positive when the stained cells were present in 10% to 50%; and positive when the stained cells were present in 50% or more.

### Western blotting analysis

Tissues were ground to power with a mortar and pestle as rapidly as possible in liquid nitrogen, washed with cold PBS, and lysed with equal volumes of RIPA lysis buffer (50 mM Tris-HCl pH 8.0, 150 mM NaCl, 0.02% NaN3, 1% Triton X-100, 1% SDS) with Cocktail protease inhibitor (1:200, Sigma, St Louis) on ice for 30 min, and then sonicated four times for 5 s each. Tissue lysates obtained following centrifugation (12,000 × g, 4°C, 10 min), were subjected to Western blotting analysis.

Protein was quantified using the Coomassie plus protein assay reagent (Pierce Chemical Co, IL) and adjusted to an equal concentration for each sample before electrophoresis. The lysates were heated in Laemmli buffer at 95°C, resolved by 8% SDS-PAGE gel (Amresco, Ohio), transferred to PVDF membrane (Roche, IN), which was then incubated with 3% BSA in Tris Buffered Saline, followed by incubation with the primary antibody against CD105 (C-20, 1:100, Santa Cruz), HIF-1alpha (1:1000, Chemicon), or beta-actin (1:1000, Boshide), and then with AP-labeled secondary antibody (1:2000, goat anti-rabbit or goat anti-mouse IgG, Santa Cruz). The signals were determined using the enhanced chemiluminance assay (NBT/BCIP Array, Huemei Bio, China).

### Measurement of tumor cytosolic VEGF_165 _protein concentration

The isolation of tumor cytosolic proteins was performed by homogenization of tissues as described by Poon [22]. Homogenates were lysed with equal volumes of RIPA lysis buffer with Cocktail protease inhibitor (1:200, Sigma) on ice for 30 minutes, and centrifuged at 12,000 rpm at 4°C for 10 minutes. VEGF_165 _in the supernatants was quantified in duplicate by ELISA Kits (Lifekey Corp.) based on a standard curve generated for each set of samples assayed. The total protein concentration was determined as described above. To correct variation caused by the different total protein concentrations, the relative level of VEGF_165 _was calculated by dividing VEGF_165 _concentration by the total protein concentration in each sample.

### Real-time PCR

Acid guanidine thiocyanate-phenol-chloroform extraction was used to isolate total RNA from tissues. With random hexamer primers, 1 μg RNA was reverse transcribed to cNDA with ExScript™ RT reagent Kits (TaKaRa, Japan). One μg RNA without reverse transcription served as negative controls. Primers and probes for human *CD105 *and *glyceraldehyde-3-phosphate dehydrogenase (GAPDH) *were designed with Primer Express 2.0 software (Applied Biosystems, Foster City, CA) and synthesized by Genecore (Shanghai, China). The basic information on the primers and probes including gene name, NCBI reference, forward primer, reverse primer, probe and its location between two exons, product size (bp) were as followings respectively: *CD105*, NM_000118, CATCCTTGAAGTCCATGTCCTCTT, GCCAGGTGCCATTTTGCTT, FAM-TCCCAACGGGCCCGTCACAG-MGB, 7 and 8, 95; *GAPDH*, NM_002046, GGGCTGCTTTTAACTCTGGTAAAG, CCATGGGTGGAATCATATTGG, FAM-CCTCAACTACATGGTTTAC-MGB, 1 and 2, 103.

Real-time PCR was performed in triplicate for each sample in a 20-μL-reaction mixture, which consisted of template DNA (2-μL), and primers (900 nM), probe (250 nM), Mg^2+ ^(5 mM), and Ex Taq HS (0.1 U/μL, ExScript™ real-time PCR Kit, TaKaRa). PCR was performed on Stratagene Mx3005P instrument using the following thermal settings: one cycle of 20 seconds at 95°C, and 55 cycles of 5 seconds at 95°C and 20 seconds at 60°C. Amplification efficiency (Eff) of each individual sample was calculated by LinRegPCR program version 7.0 (a gift from Prof. Pfaffl, Academic Medical Centre, University of Amsterdam, Amsterdam, the Netherlands). According to the method tested by Tichopad [23], the relative expression ratio (RR) of *CD105 *gene was calculated based on Eff and the Ct comparative with a reference gene (*GAPDH*) in a sample.

### Statistical analysis

Data were expressed as mean ± standard deviation with the range given in parentheses. Statistical analysis was performed using the *t *tests, ANOVA and linear regression when data were normally distributed. The Pearson χ^2 ^test was used to compare the results of two or more subgroups. All statistical procedures were performed using SPSS (Version 11.5 SPSS Inc, Chicago). Values of *p *< 0.05 were considered as statistically significant.

## Results

### Characteristics of the HCC patients

In 64 patients (53 males and 11 females, median age 51 yrs) who underwent curative resection (57 cases for regular hepatectomy and 7 for orthotopic liver transplantation), the average tumor size was 6.65 ± 4.17 cm (range: 0.8–20 cm). There were 40 patients with large tumor (maximum diameter > 5 cm), 24 with small tumor (maximum diameter ≤ 5 cm), and 22 with multiple tumors (more than two lesions). Liver cirrhosis was detected in 60 patients, and the remaining 4 patients had chronic hepatitis. The etiologies of underlying liver diseases were hepatitis B in 56 patients, hepatitis C in 1, mixed viral infection in 1, alcoholic cirrhosis in 4, and cirrhosis of unidentified etiology in 3 patients. According to UICC recommendations (2002) [19], 25 patients were classified as stage I, 12 patients as stage II, 26 patients as stage III, and 1 patient as stage IV. Fifty-two patients were in Child's class A, 11 in class B, and 1 in class C.

### Distribution and expression of CD105 in liver with HCC: CD105 rich in non-tumor tissues

**Figure 1:**
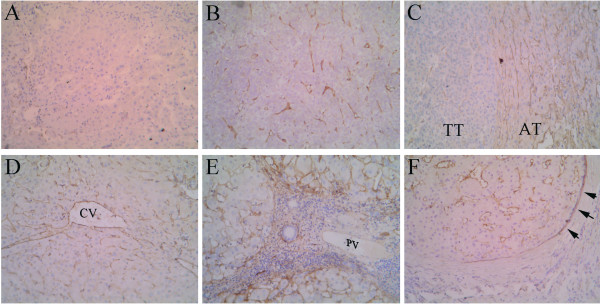
**Distribution of CD105 antigen in normal, non-tumor (TF and AT), and tumor tissues**. *A*, Immunostaining for CD105 in normal tissues. *B*, Highlighted microvessels by CD105 in tumor tissues. *C*, Diffuse positive sinusoidal segments found in the surrounding of tumor. *D*, Diffuse positive HSECs in the surrounding of draining veins (CV, central vein) in non-tumor tissues. *E*, Distribution of CD105 in portal area (PV, portal vein) in non-tumor tissues. *F*, CD105-positive septal fibroblasts (black arrows) in periphery of pseudolobules in non-tumor tissues. The signals were detected by DAB staining. Magnification: ×200.

**Figure 2:**
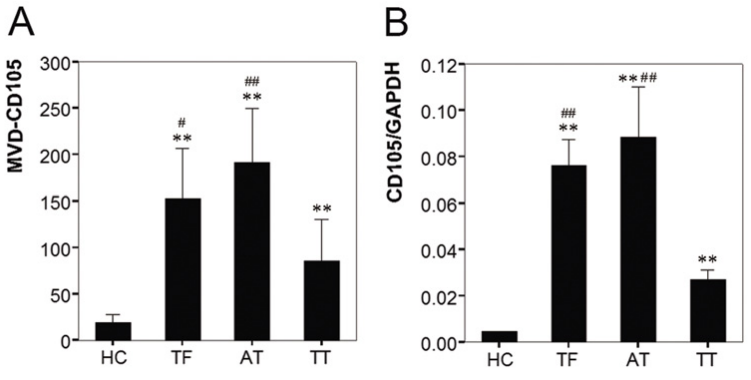
**MVD-CD105 and CD105 mRNA level in normal, non-tumor (TF and AT), and tumor tissues**. *A*, MVD-CD105 in HC (*n *= 8), TF, AT, and TT (*n *= 64); *B*, Relative level of *CD105 *mRNA in HC (*n *= 8), TF, AT, and TT (*n *= 64) (* = *p *< 0.05, ** = *p *< 0.01, versus HC; ^# ^= *p *< 0.05, ^## ^= *p *< 0.01, versus TT); Columns, mean; bars, SD.

In normal liver tissues, expression of the CD105 antigen was restricted to only a few hepatic sinus endothelial cells (HSECs) located in the direct vicinity of portal tracts (Figure 1A). Similar to Ho's report [17], highlighted microvessels by CD105 showed three patterns of expression in the tumor tissue sections: sinusoid-like, branching, and small without apparent lumina (endothelial sprouts, Figure 1B and 1C). However, the expression pattern of CD105 in AT and TF was different from that in TT in the following aspects. First, CD105 showed a diffuse pattern of staining in most cases (51/64), predominantly on HSECs in the surrounding of draining veins (Figure 1C and 1D). Second, there were no CD105 positive cells in portal veins, hepatic arteries or biliary ducts (Figure 1E). Third, besides in HSECs, some CD105 positive cells, such as on septal fibroblasts, existed in the surrounding of pseudolobules in focal nodular hyperplasia (Figure 1F). Furthermore, the mean score of MVD-CD105 (mean ± SD/0.74 mm^2^) was 19.00 ± 9.08 in HC, 153.12 ± 53.26 in TF, 191.12 ± 59.17 in AT, and 85.43 ± 44.71 in TT, respectively. MVD-CD105 in HC was significantly lower than in TF, AT, and TT (One-Way ANOVA, *p *< 0.001). Paired *t *test showed that MVD-CD105 in TF and AT was significantly higher than in TT (*p *= 0.012 and *p *= 0.007, respectively) while there was no significant difference between TF and AT (Figure 2A).

**Figure 3:**
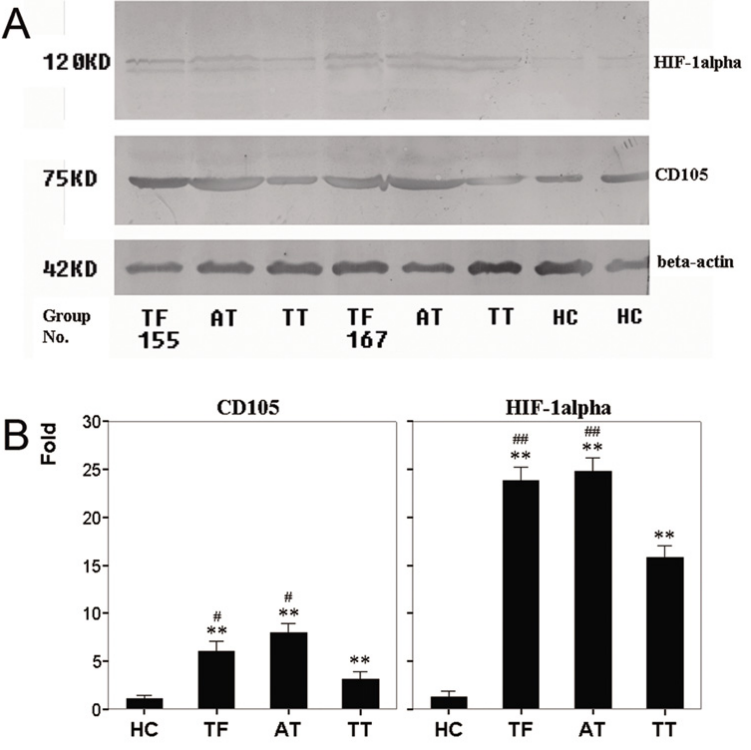
**Relative Expression of CD105 and HIF-1alpha antigens in normal, non-tumor (TF and AT), and tumor tissues**. CD105 and HIF-1alpha antigens were detected by Western blotting analysis in 16-paired samples, and the representative data were presented. *A*, Proteins in lanes 1–3 were extracted from TF, AT, and TT of patient 155, respectively; lanes 4–6 from TF, AT, and TT of patient 167, and lanes 7 and 8 from normal tissues of two healthy controls. *B*, Fold of CD105 and HIF-1alpha represented the mean of the relative fold from 8 independent experiments (HC, *n *= 8; TF, AT, and TT, *n *= 16). Relative fold refers to the ratio of CD105 or HIF-1alpha intensity in TF, AT, and TT to that in HC (* = *p *<0.05, ** = *p *< 0.01, versus HC; ^# ^= *p *< 0.05, ^## ^= *p *< 0.01, versus TT). Columns, mean; bars, SD.

Previous investigations reported that all antibodies against CD105 did not show the same specificity [1]. To examine whether different antibodies to CD105 resulted in varied immunohistochemical staining, we evaluated the mRNA level of *CD105 *in 64 paired specimens by real-time PCR and CD105 antigen in 16 paired samples by Western blots with another CD105 antibody (C-20). The relative level of *CD105 *mRNA (*CD105*/*GAPDH*, mean ± SD) was 0.0045 ± 0.0009 in HC, 0.076 ± 0.011 in TF, 0.088 ± 0.021 in AT, and 0.029 ± 0.005 in TT, respectively. *CD105 *mRNA in HC was significantly lower than in TF, AT, and TT (One-Way ANOVA, *p *= 0.001). Paired *t *test showed that the *CD105 *mRNA in AT and TF was significantly higher than in TT (*p *< 0.001 and *p *= 0.009, respectively) and there was no significant difference between TF and AT (Figure 2B). Moreover, Western blotting analysis revealed that the CD105 levels in TF, AT, and TT were 5.02-, 6.98- and 2.14-fold higher than that in HC (*n *= 8, *p *< 0.001), respectively, and CD105 in TF and AT were significantly higher than in TT (*p *= 0.038 and *p *= 0.027, respectively; Figure 3).

Consequently it was demonstrated at protein and mRNA levels that the CD105 was expressed mostly on HSECs and microvessels, more abundantly in AT and TF than in TT.

### Coincident distribution and expression of HIF-1alpha with CD105

**Figure 4:**
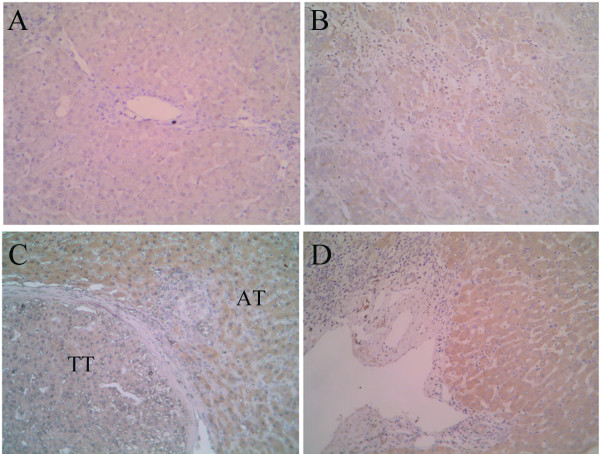
**Distribution of HIF-1alpha antigen in normal, non-tumor (TF and AT), and tumor tissues**. Immunostaining for HIF-1alpha in HC (*A*), tumor tissues (*B*), the surrounding of tumor (*C*), and portal area of non-tumor tissues (*D*). The signals were detected by DAB staining. Magnification: ×200.

The levels of CD105 protein, mRNA and promoter activity can be up-regulated by hypoxia via the HIF-1 complex, which binds a functional consensus HRE in the endoglin promoter [24]. The distribution and expression of HIF-1alpha in liver tissues with HCC were evaluated by Immunohistochemistry and Western blotting analysis. The positive staining was located in the cytoplasm and/or the nuclei of tumor cells and hepatocytes (Figure 4B–D). In general, the intensity of HIF-1alpha staining in the non-tumor tissues (TF and AT) was higher than in tumor tissues (Figure 4C). In the portal area of cirrhosis, the expressions of HIF-1alpha in the bile duct and the vessels were negative (Figure 4D). Among the 64 paired specimens, HIF-1alpha was expressed in 88.13% of TT, which was lower than in AT (96.46%) and TF (92.39%), but was higher than that in normal hepatic tissues (zero, Figure 4A). In accordance with the results of immunohistochemical staining, Western blotting analysis revealed that the HIF-1alpha levels in TF, AT, and TT were 22.82-, 23.81-, and 14.79-fold higher than that in HC, respectively (*n *= 8, *p *< 0.001), and the HIF-1alpha levels in TF and AT were significantly higher than in TT (*p *= 0.006 and *p *= 0.001, respectively; Figure 3).

### Correlated distribution and expression of VEGF_165 _with CD105

**Figure 5:**
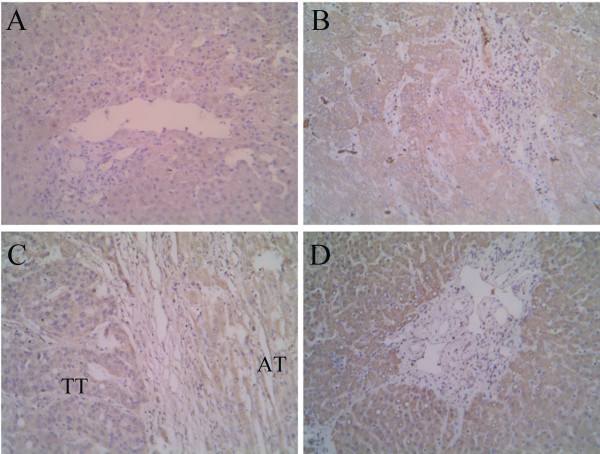
**Distribution of VEGF antigen in normal, non-tumor (TF and AT), and tumor tissues**. Immunostaining for VEGF in HC (*A*), tumor tissues (*B*), the surrounding of tumor (*C*), and portal area of non-tumor tissues (*D*). The signals were detected by DAB staining. Magnification: ×200.

**Figure 6:**
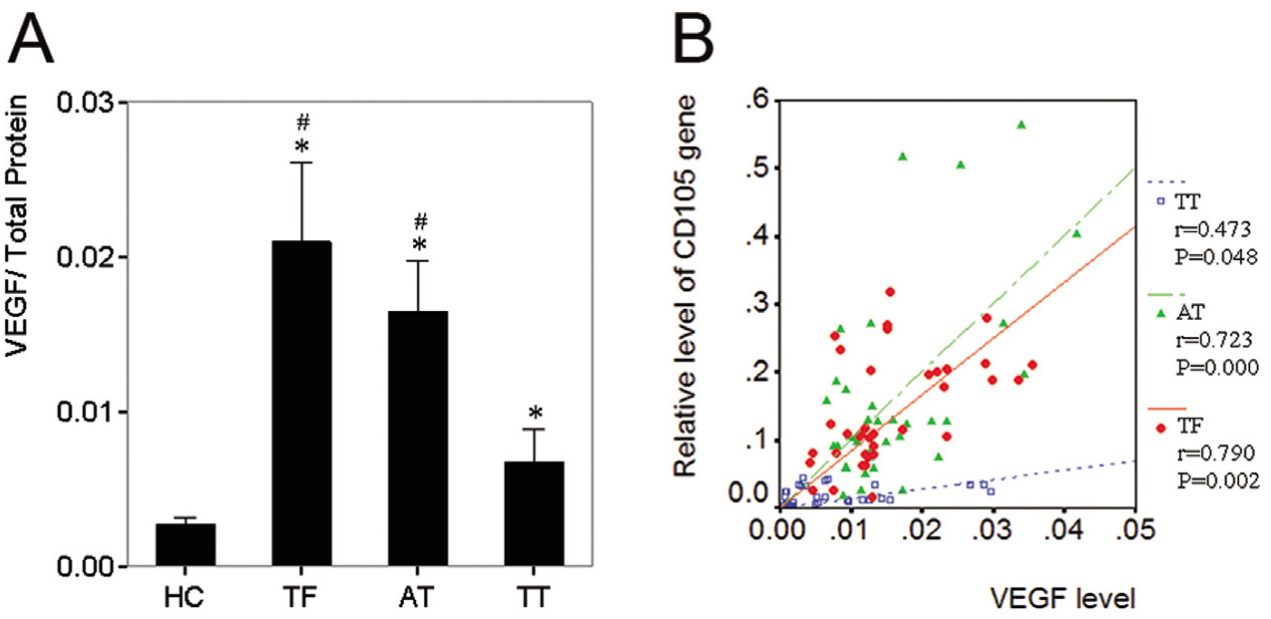
**Relative level of VEGF in normal, non-tumor (TF and AT), and tumor tissues and the correlation of VEGF and CD105**. *A*, Relative level of VEGF in HC (*n *= 8), TF, AT, and TT (*n *= 36, * p < 0.05 versus HC; # p < 0.05 versus TT). Columns, mean; bars, SD. *B*, Correlation between VEGF and CD105 mRNA in TF, AT, and TT (*n *= 36).

Previous clinical investigations reported that CD105 correlated with VEGF_165 _in some tumors, such as non-small cell lung cancer [5], HCC [25], and breast cancer [26]. The distribution and expression of VEGF_165 _in liver tissues with HCC were evaluated by Immunohistochemistry and ELISA. The positive staining of VEGF_165 _mainly existed in cytoplasm of tumor cells and hepatocytes (Figure 5B–D). In general, the intensity of VEGF_165 _staining in the non-tumor tissues (TF and AT) was higher than in tumor tissues (Figure 5C). VEGF_165 _signals were also present in endothelial cells (Figure 5D). Among the 64 paired specimens, VEGF_165 _was positively stained in 37.12% of TT, which was lower than in AT (56.23%) and TF (47.91%), but was higher than in normal hepatic tissues (zero, Figure 5A). In addition, ELISA analysis also revealed that VEGF_165 _protein in HC was significantly lower than in TF, AT, and TT (One-Way ANOVA, *p *= 0.017). Paired *t *test showed that VEGF_165 _protein in TF and AT was significantly higher than in TT (*n *= 36, *p *= 0.025, and *p *= 0.024, respectively; Figure 6A). Paired correlation analysis showed that *CD105 *mRNA correlated with VEGF_165 _in TF (r = 0.790, *p *= 0.002), AT (r = 0.723, *p *< 0.001), and TT (r = 0.473, *p *= 0.048), respectively (Figure 6B).

## Discussion

Previous reports of Ho's and ours have demonstrated that CD105 is a better prognostic marker for HCC than MVD assessed by a pan-endothelial cell marker CD34 [16,17]. However, our present study showed that CD105 had lower expression in tumor tissues than in non-tumor tissues with cirrhosis, in which the expression of CD105 antigen was found mostly in HSECs, especially in outflow area of AT and TF. In agreement with our findings, Theuerkauf et al [27] revealed the common character of the distribution of CD105 in liver with three different pathological conditions (localized increased perfusion, chronic congestion, decreased portovenous or arteriohepatic perfusion): CD105 immunoreactivity was mostly restricted to HSECs while that in portal blood vessels was negative. An increased CD105 expression on HSECs and septal fibroblasts in non-tumor tissues of patients with chronic liver diseases has been observed [28]. Moreover, Ho's investigation showed that the disease-free survival in patients with a diffuse pattern of CD105 staining in AT was statistically worse than that in patients without a diffuse pattern of CD105 expression [17]. However, Yang et al reported that CD105 was not expressed in the vascular endothelial cells of the paracarcinomatous liver tissues in any of the 113 specimens [25]. Although different antibodies to CD105 antigen may result in varied expression of CD105 in liver with HCC, as well as some other types of cancer [29-33], the discrepancy of our findings and Yang's may not be caused by the different antibodies to CD105 antigen (see below).

To clarify whether above discrepancy was caused by different antibodies, we evaluated the protein level of CD105 in three parts of the specimens from HCC by two different CD105 antibodies (H-300 and 4C11) and mRNA level by real-time PCR in the present investigation. All of the above results showed the similar distribution and expression. To validate the specificity of antibodies, we further evaluated the distribution and expression of CD105 in ductal infiltrative breast cancer, colon cancer, renal cancer, and their surrounding tissues using above two antibodies. Of note, there was a significant correlation between microvessel counts stained by two CD105 antibodies in breast, colon and renal cancer samples (Figure 2, 3, 4 in Supplementary Data). In addition, we studied the distribution and expression of CD105 by H-300 and 4C11 antibodies in our own HCC tissue arrays developed in 2006. The results displayed the same distribution and expression of CD105 in two tissue array sections (Figure 5 in Supplementary Data). Therefore, the discrepancy in immunohistochemical staining with different antibodies was excluded.

The molecular basis for CD105 up-regulation is not completely defined, but there is increasing experimental evidence that hypoxia can stimulate *CD105 *mRNA expression in vascular endothelial cells via the HIF-1 complex, which binds a functional consensus HRE in the endoglin promoter [24]. As a key transcript factor under hypoxia, HIF-1alpha had higher expression in non-tumor cirrhotic tissues than in tumor tissues in this study (Figure 3 and 4B–D). The distribution and expression of CD105 in liver with HCC was consistent with those of HIF-1alpha. In a further investigation in 13 cirrhotic liver tissues (CT), the expression pattern of CD105 in CT was similar to that in AT and TF (Figure 1 in Supplementary Data). In this regard, cirrhosis might induce hypoxia condition in liver tissues. The non-tumor tissues themselves have precancerous changes with angiogeneses [34]. During liver cirrhosis, fibrogenesis induces intrahepatic shunts and the barrier between the sinusoids and the hepatocytes [35]. Fibrous pseudo lobes form as discrete hypoxia unit to induce angiogenesis [34]. Furthermore, hepatitis B virus X protein increases the transcriptional activity and protein level of HIF-1alpha, and thereby promote angiogenesis during hepatocarcinogenesis [36]. Therefore cells in cirrhotic liver are under a sustained, mechanically reduced blood flow, which induces angiogenesis in cirrhotic tissues [14].

It has been documented that CD105 correlates with VEGF_165 _in some tumors, such as non-small cell lung cancer [5], HCC [25], and breast cancer [26]. In this study, we found that intrahepatic CD105 correlated with VEGF_165 _in both tumor tissues and non-tumor tissues (Figure 6). The fact that decreased oxygen pressure is a strong stimulus for VEGF transcription and protein synthesis may explain the up-regulation of the VEGF in the cirrhotic liver [37,38]. Moreover, VEGF expression is also modulated by cytokines released from the infiltrating inflammatory cells in surrounding cirrhotic liver tissues [39]. Therefore it is widely accepted that pro-angiogenic factors, such as VEGF_165 _[14,18,39] and HGF [40], have higher expression in the surrounding tissues than in tumor tissues. We further evaluated the distribution and expression of CD105 in 13 cirrhotic liver tissues (CT). The expression pattern of CD105 in CT was similar to that in TF and AT from HCC (Figure 1 in Supplementary Data). Taken together, cirrhosis might induce hypoxia and the expression of some pro-angiogenic factors in cirrhotic tissues, and modulate the expression of CD105 in turn. Of note, MVD in AT is higher than that in any of HC, CT, TF, and TT. Therefore, the CD105 over-expression in the tumor free tissue may be a reflection of both cirrhosis and a "field effect" relevant to the tumor. It is worthy investigating the relevant mechanism further.

Because of the particular distribution and expression of CD105 in liver with HCC, targeting for antiangenesis therapy with CD105 antibodies in HCC should be considered cautiously. Because (1) increased CD105 expression was not only on endothelial cells in tumor neovessels, but also on HSECs and septal fibroblasts in non-tumor tissues; (2) CD105 immunoreactivity was mostly restricted to the endothelium of draining veins; (3) CD105 is a cell surface antigen widely expressed on vascular endothelium, syncytiotrophoblast, some tissue macrophages, and progenitor cells [41]. Even though the staining in normal mucosa was weaker than in tumor tissues, all tissues were found to be positive, at least in microvessels, except for normal breast. Moreover, a positive reaction was observed in the stroma of some tissues (glands and reproductive tract) [42]. Because of above concerns, targeting with CD105 in HCC lacks of specificity, may result in fetal side effects, such as hemorrhage, liver dysfunction, and interfere with physiological angiogenesis.

## Conclusion

Our data showed that the expression of *CD105 *at mRNA and protein levels was higher in tumor tissues than in normal liver, but was lower than in non-tumor tissues with cirrhosis. Noticeably, CD105 showed a diffuse pattern of staining predominantly on HSECs in non-tumor tissues. Therefore, CD105 might not be an appropriate targeting for antiangenesis therapy in HCC with liver cirrhosis. In addition, the presence of well-diffuse patterns of CD105 expression in the adjacent non-tumor tissues could predict its key role during cirrhosis. Further studies are merited to clarify the mechanisms involved in cirrhosis.

## Competing interests

The author(s) declare that they have no competing interests.

## Authors' contributions

DCY participated in its design, carried out real-time PCR and Western blotting analysis, and wrote the paper. LYZ collected all of specimens and clinical database, and carried out Western blotting analysis. XTS participated in the design of the study and helped to draft the manuscript. JC and KM carried out the immunoassays and analyzed the results. YZY participated in its design. YTD conceived of the study, participated in its design, and give final approval of the version to be published. All authors read and approved the final manuscript.

## Pre-publication history

The pre-publication history for this paper can be accessed here:



## Supplementary Material

Additional file 1 Supplementary Data. The data represent all annotation, results, and legends of the supplementary data referred in Disscussion section. Click here for file

Additional file 2 CD105 expression in cirrhotic liver tissues (Figure 1 in Supplementary Data). This figure shows the distribution and expression of CD105 expression in cirrhotic liver tissues. Click here for file

Additional file 3 CD105 expression in breast cancer tissues (Figure 2 in Supplementary Data). This figure shows the representative data on the expression of CD105 in tumor tissues and tumor free tissues from breast cancer samples stained by 4C11 and H300 CD105 antibodies. Click here for file

Additional file 4 CD105 expression in colon cancer tissues (Figure 3 in Supplementary Data). This figure shows the representative data on the expression of CD105 in tumor tissues and tumor free tissues from colon cancer samples stained by 4C11 and H300 CD105 antibodies. Click here for file

Additional file 5 CD105 expression in renal cancer tissues (Figure 4 in Supplementary Data). This figure shows the representative data on the expression of CD105 in tumor tissues and tumor free tissues from renal cancer samples stained by 4C11 and H300 CD105 antibodies. Click here for file

Additional file 6 CD105 expression in HCC tissue arrays (Figure 5 in Supplementary Data). This figure shows the representative data on the expression of CD105 in normal tissues, cirrhotic tissues, tumor free tissue, and tumor tissues dotted on two pieces of HCC tissue array. Click here for file
