# Supplementary material for: Particular distribution and expression pattern of endoglin (CD105) in the liver of patients with hepatocellular carcinoma
Source: BMC Cancer. 2007 Jul 4;7:122. doi: 10.1186/1471-2407-7-122 (PMC1941740; doi:10.1186/1471-2407-7-122)
Supplement: Additional file 1 — Supplementary Data. The data represent all annotation, results, and legends of the supplementary data referred in Disscussion section. [file 1471-2407-7-122-S1.doc]

# Supplementary Data

### For Particular distribution and expression pattern of endoglin (CD105) in the liver of patients with hepatocellular carcinoma. DC Yu et al.

To determine in fact if the CD105 over-expression in the benign tissue is a reflection of cirrhosis or a “field effect” related to the tumor, we further evaluated the distribution and expression of CD105 in 13 cirrhotic liver tissues (CT). The expression pattern of CD105 in CT was similar to that in TF and AT from HCC in the following aspects. First, CD105 showed a diffuse pattern of staining in most cases (10/13), predominantly on HSECs in the surrounding of draining veins (Figure 1A). Second, there were no CD105 positive cells in portal veins, hepatic arteries or biliary ducts (Figure 1B). Third, besides in HSECs, some CD105 positive cells, such as on septal fibroblasts, existed in the surrounding of pseudolobules in focal nodular hyperplasia (Figure 1C). Furthermore, the mean score of MVD-CD105 (Mean ± SD/0.74 mm^2^) was 167.58 ± 44.86, which was significantly higher than those in HC and TT, but was in the similar level as those in TF and AT (Figure 1D). Of note, MVD in AT was the highest among HC, CT, TF, AT, and TT. Therefore the CD105 overexpression in the tumor free tissue might be a reflection of both cirrhosis and a “field effect” relevant to the tumor. It is worthy investigating further the relevant mechanism.

To validate the specificity of antibodies, we further evaluated the distribution and expression of CD105 in ductal infiltrative breast cancer, colon cancer, renal cancer, and their tumor free tissues (TF) (*n* = 3) using two antibodies (H-300 and 4C11). CD105 expression was intense and restricted to capillary endothelial cells in tumor tissues (TT). The results showed that MVD-CD05 was higher in TT from breast cancer and colon cancer than in TF, which was consistent with the results from previous investigations (Figure 2 and 3, and see below Reference 1 and 2). However, there was no significant difference in CD105 expression between TT and TF from renal cancer samples, which was consistent with the results from the investigation by Minhajat (Figure 4 and see below Reference 3). Of note, there was a significant correlation between microvessel counts stained by two CD105 antibodies in breast, colon, and renal cancer samples [Figure 2F, 3F, and 4F].

To examine whether different antibodies to CD105 resulted in varied immunohistochemical staining, we further evaluated the distribution and expression of CD105 antigen by CD105 antibodies (H300 and 4C11) in our own tissue arrays developed in 2006, including 25 normal liver from healthy controls (HC), 19 cirrhotic tissues (CT), 113 pair of TT and AT samples, as described by Yao, et al (See below Reference 4). After immunohistochemical staining, the number in the different groups of samples was summarized in Table 1 according to the staining intensity by two antibodies. For CD105 staining of MVD (MVD-CD105), the categories were as follows: negative, 0; positive, < 60; and diffuse positive >60. There was significant difference among HC, CT, AT, and TT. Of note, the results from tissue arrays using two different antibodies revealed the similar distribution and expression of CD105 in two tissue array sections (Figure 5 and Table 1).

### Reference

1. Bodey B, Bodey B Jr, Siegel SE, Kaiser HE: **Over-expression of endoglin (CD105): a marker of breast carcinoma-induced neo-vascularization.** *Anticancer Res.* 1998, 18:3621-3628.
2. Li C, Gardy R, Seon BK, Duff SE, Abdalla S, Renehan A, O'Dwyer ST, Haboubi N, Kumar S: **Both high intratumoral microvessel density determined using CD105 antibody and elevated plasma levels of CD105 in colorectal cancer patients correlate with poor prognosis.** *Br J Cancer.* 2003, 88:1424-1431.
3. Minhajat R, Mori D, Yamasaki F, Sugita Y, Satoh T, Tokunaga O: **Organ-specific endoglin (CD105) expression in the angiogenesis of human cancers.** *Pathol Int.* 2006, 56:717-723.
4. Yao YZ, Pan YM, Chen J, Sun XT, Qiu YD, Ding Yitao: **Endoglin (CD105) expression in angiogenesis of primary hepatocellular carcinoma：analysis using tissue microarray and comparison with CD34 and VEGF.** *Ann Clin Lab Sci* 2007, 37:39-48.

### Figure 1 Immunohistochemical analysis of CD105 expression in cirrhotic liver tissues

*A-C*, Representative data on immunostaining for CD105 in cirrhotic tissues (The signals were detected by DAB staining. Magnification: ×200). *D*, MVD-CD105 in HC (*n* = 8), CT (*n* = 13), TF, AT, and TT (*n* = 64) is compared using *t* test (* = ***p*** < 0.05, ** = ***p*** < 0.01, versus HC; # = ***p*** < 0.05, ## = ***p*** < 0.01, versus TT); Columns, mean; bars, SD.

### Figure 2 Immunohistochemical analysis of CD105 expression in tumor tissues and tumor free tissues from breast cancer samples

Representative data on the expression of CD105 in TF (*A* and *B*) and TT (*C* and *D*) stained by 4C11 (*A* and *C*) and H300 CD105 (*B* and *D*) antibodies, are shown (The signals were detected by DAB staining. Magnification: ×200). *E* and *F*, MVD in TT and TF are compared with paired *t* tests (* = ***p*** < 0.05, versus AT); Columns, mean; bars, SEM.

### Figure 3 Immunohistochemical analysis of CD105 expression in tumor tissues and tumor free tissues from colon cancer samples

Representative data on the expression of CD105 in TF (*A* and *B*) and TT (*C* and *D*) stained by 4C11 (*A* and *C*) and H300 CD105 (*B* and *D*) antibodies, are shown (The signals were detected by DAB staining. Magnification: ×200). *E* and *F*, MVD in TT and TF are compared with paired *t* tests (* = ***p*** < 0.05, versus AT); Columns, mean; bars, SEM.

### Figure 4 Immunohistochemical analysis of CD105 expression in tumor tissues and tumor free tissues from renal cancer samples

Representative data on the expression of CD105 in TF (*A* and *B*) and TT (*C* and *D*) stained by 4C11 (*A* and *C*) and H300 CD105 (*B* and *D*) antibodies, are shown (The signals were detected by DAB staining. Magnification: ×200). *E* and *F*, MVD in TT and TF are compared with paired *t* tests (* = ***p*** < 0.05, versus AT); Columns, mean; bars, SEM.

### Figure 5 Immunohistochemical expression of CD105 on HCC tissue arrays

Immunohistochemical expression of CD105 on tissue arrays is shown using 4C11 (*A*, *C*, *E* and *G*) and H300 (*B*, *D*, *F* and *H*) antibodies. Representative data on the expression of CD105 in normal tissues (*A* and *B*), cirrhotic tissues (*C* and *D*), tumor free tissue (*E* and *F*) and tumor tissues (*G* and *H*), are presented. The signals were detected by DAB staining. Magnification: ×200.

### Table 1. The number of the samples of different staining intensity in HC, CT, AT, and TT

| **Antibodies** | | | **Samples** | | | | **Total** | ***p1*** |
| --- | --- | --- | --- | --- | --- | --- | --- | --- |
|  |  |  | **HC** | **CT** | **AT** | **TT** |  |  |
| 4C11 | Intensity | Negative | 10 | 1 | 4 | 9 | 24 | < 0.01 |
|  |  | Positive | 15 | 5 | 28 | 83 | 131 |  |
|  |  | Diffuse positive | 0 | 13 | 81 | 21 | 115 |  |
|  | Total | | 25 | 19 | 113 | 113 | 270 |  |
| H300 | Intensity | Negative | 12 | 1 | 5 | 11 | 29 | < 0.01 |
|  |  | Positive | 13 | 3 | 25 | 76 | 117 |  |
|  |  | Diffuse positive | 0 | 15 | 83 | 26 | 124 |  |
|  | Total | | 25 | 19 | 113 | 113 | 270 |  |
| ***p2*** | | | 0.569 | 0.725 | 0.858 | 0.594 |  |  |

*(p1: Chi-Square Tests for the samples; p2: 4C11 versus H300 in the different groups of samples)*
