# Supplementary figures and images for: Particular distribution and expression pattern of endoglin (CD105) in the liver of patients with hepatocellular carcinoma
Source: BMC Cancer. 2007 Jul 4;7:122. doi: 10.1186/1471-2407-7-122 (PMC1941740; doi:10.1186/1471-2407-7-122)

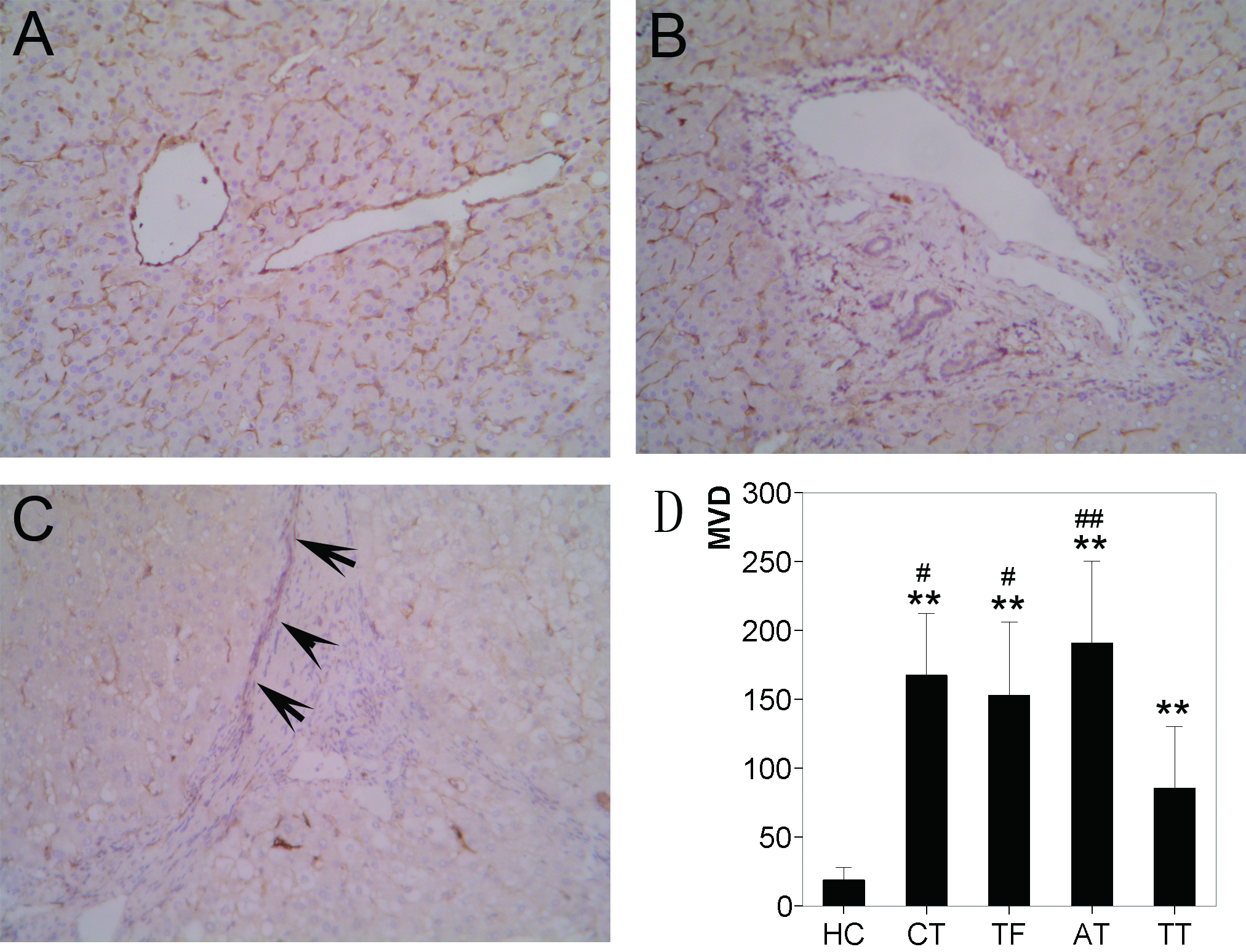

Supplement: Additional file 2 — CD105 expression in cirrhotic liver tissues (Figure 1 in Supplementary Data). This figure shows the distribution and expression of CD105 expression in cirrhotic liver tissues. [file 1471-2407-7-122-S2.jpeg]

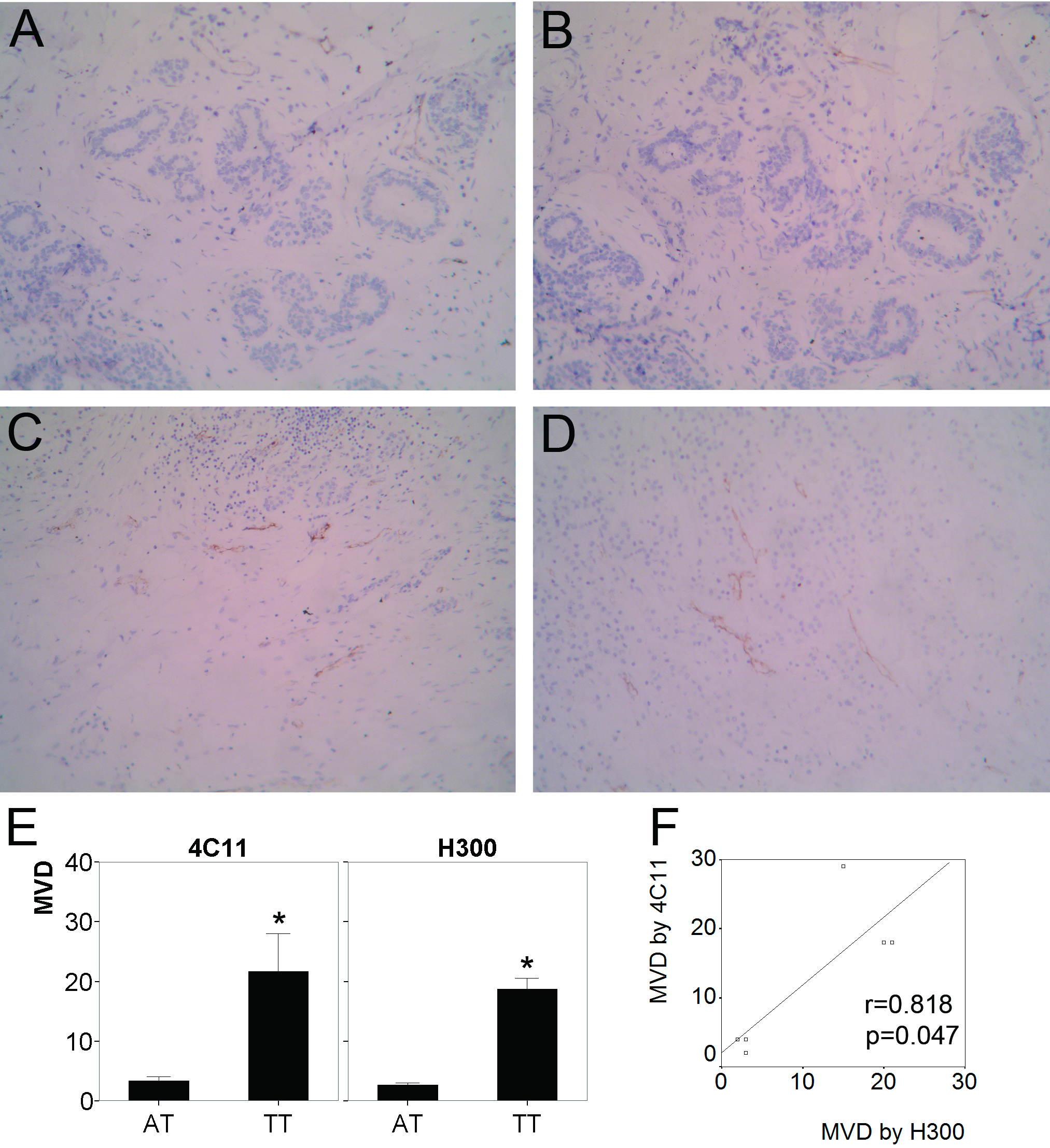

Supplement: Additional file 3 — CD105 expression in breast cancer tissues (Figure 2 in Supplementary Data). This figure shows the representative data on the expression of CD105 in tumor tissues and tumor free tissues from breast cancer samples stained by 4C11 and H300 CD105 antibodies. [file 1471-2407-7-122-S3.jpeg]

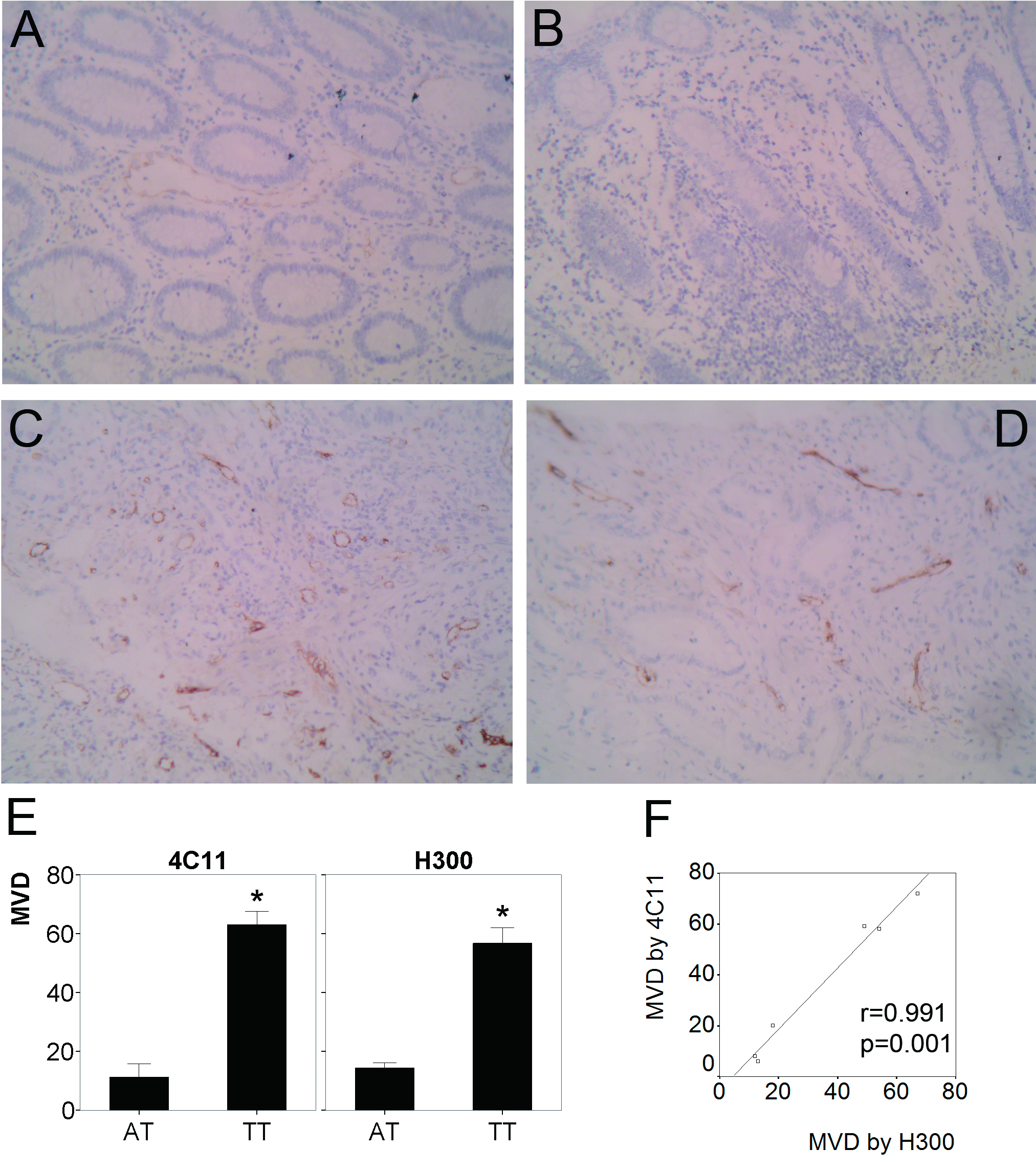

Supplement: Additional file 4 — CD105 expression in colon cancer tissues (Figure 3 in Supplementary Data). This figure shows the representative data on the expression of CD105 in tumor tissues and tumor free tissues from colon cancer samples stained by 4C11 and H300 CD105 antibodies. [file 1471-2407-7-122-S4.jpeg]

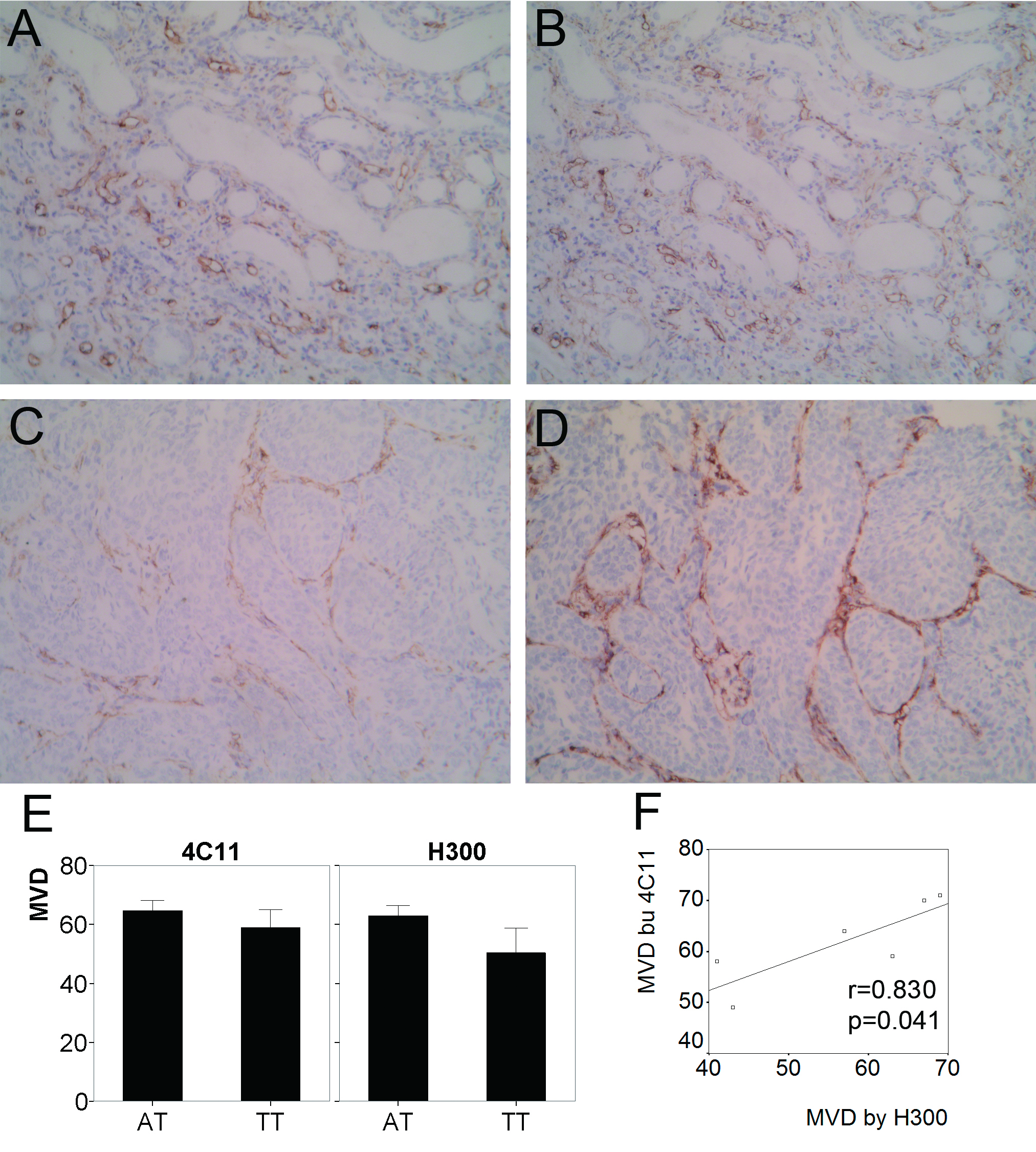

Supplement: Additional file 5 — CD105 expression in renal cancer tissues (Figure 4 in Supplementary Data). This figure shows the representative data on the expression of CD105 in tumor tissues and tumor free tissues from renal cancer samples stained by 4C11 and H300 CD105 antibodies. [file 1471-2407-7-122-S5.jpeg]

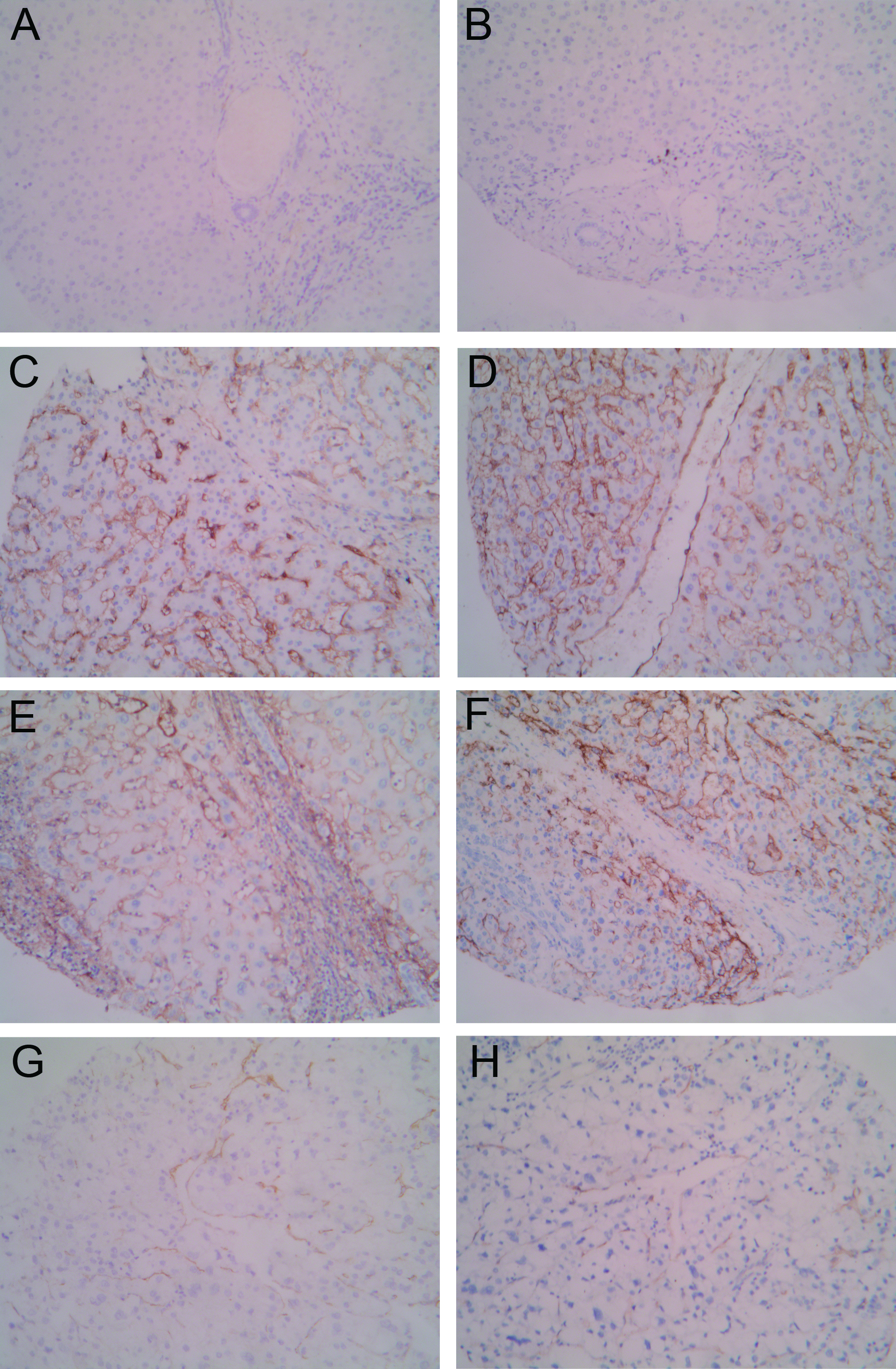

Supplement: Additional file 6 — CD105 expression in HCC tissue arrays (Figure 5 in Supplementary Data). This figure shows the representative data on the expression of CD105 in normal tissues, cirrhotic tissues, tumor free tissue, and tumor tissues dotted on two pieces of HCC tissue array. [file 1471-2407-7-122-S6.jpeg]
